# The Projection of Burden of Disease in Islamic Republic of Iran to 2025

**DOI:** 10.1371/journal.pone.0076881

**Published:** 2013-10-17

**Authors:** Razieh Khajehkazemi, Behnam Sadeghirad, Mohammad Karamouzian, Mohammad-Sadegh Fallah, Mohammad-Hossien Mehrolhassani, Reza Dehnavieh, AliAkbar Haghdoost

**Affiliations:** 1 Regional Knowledge Hub, and WHO Collaborating Centre for HIV Surveillance, Institute for Futures Studies in Health, Kerman University of Medical Sciences, Kerman, Iran; 2 Neuroscience Research Center, Institute of Neuropharmacology, Kerman University of Medical Sciences, Kerman, Iran; 3 Kawsar Human Genetics Research Center, Tehran, Iran; 4 Research Center for Health Services Management, Department of Health Services Management, Institute for Futures Studies in Health, Kerman University of Medical Sciences, Kerman, Iran; 5 Research Center for Modeling in Health, Institute for Futures Studies in Health, Kerman University of Medical Sciences, Kerman, Iran; Kenya Medical Research Institute - Wellcome Trust Research Programme, Kenya

## Abstract

**Objective:**

Iran as a developing country is in the transition phase, which might have a big impact on the Burden of Disease and Injury (BOD). This study aims to estimate Burden of Disease and Injury (BOD) in Iran up to 2025 due to four broad cause groups using Disability-Adjusted Life Year (DALY).

**Methods:**

The impacts of demographic and epidemiological changes on BOD (DemBOD and EpiBOD) were assessed separately. We estimated DemBOD in nine scenarios, using different projections for life expectancy and total fertility rate. EpiBOD was modeled in two scenarios as a proportion of DemBOD, based on the extracted parameters from an international study.

**Findings:**

The BOD is projected to increase from 14.3 million in 2003 to 19.4 million in 2025 (95% uncertainty interval: 16.8, 21.9), which shows an overall increase of 35.3%. Non-communicable diseases (12.7 million DALY, 66.0%), injuries (4.6 million DALY, 24.0%), and communicable diseases, except HIV/AIDS (1.8 million DALY, 9%) will be the leading causes of losing healthy life. Under the most likely scenario, the maximum increase in disease burden due to DemBOD is projected to be observed in HIV/AIDS and Non-communicable diseases (63.9 and 62.4%, respectively) and due to EpiBOD in HIV/AIDS (319.5%).

**Conclusion:**

It seems that in the following decades, BOD will have a sharp increase in Iran, mainly due to DemBOD. It seems that communicable diseases (except HIV/AIDS) will have less contribution, and especially non-communicable diseases will play a more significant role.

## Introduction

Burden of Disease (BOD) is a population health index which measures the gap between current health status and an ideal situation where one lives into old age in full health, free of disease and disability, using Disability-Adjusted Life Year (DALY). The DALY is a sum of years of life lost due to premature death (YLL) and period lived with disabilities (YLD). Therefore, one DALY is considered as one lost year of healthy life due to disability or death [1].

Projection of future BOD can be used as evidence for health policy makers to assess the nature and extent of health problems in a population and set up plans for them [2]–[3]. Studies have been carried out to project the Global Burden of Disease (GBD) [2], [4]. In addition to GBD, several studies in developed countries such as France, Australia, and Western Australia have also projected the BOD [5]–[7]. No such study was found in developing countries, which is likely due to various issues in different healthcare system such as the inaccuracy or lack of death registration data, cause-of-death information, periodical data on disease specific prevalence and incidence, and finally, missing information regarding disability and remission durations of each disease or injury [8]–[10].

In this study, we modeled BOD until 2025 in Iran and assessed its changes between 2003 and 2025. Iran is a developing country located in the Middle East with a population of more than 70 million people. The World Bank places it geographically in the Middle East and North Africa and economically among upper-mid income countries (Per Capita Gross National Income was US $4590 in 2009) [11].

There are a few considerations in the estimation of BOD in Iran. On the national level, there is only one estimation for BOD [12]; therefore it is not easy to model its time-trend. Moreover, we could not find any consistent information about the long-term trend of many epidemiological indices which are usually used in the modeling of BOD [2].

Based on the above explanation, we tried to project the BOD in Iran up to 2025. However, because of these limitations, we assumed that the total changes in BOD are due to the impact of demographic (changes in size and age-sex structure of a population) and epidemiological (changes in the prevalence and incidence of diseases and their risk factors) components.

## Materials and Methods

To project the BOD in Iran in 2025 and its changes from 2003 up to 2025, due to the impact of demographic and epidemiological factors, we performed our analysis in six steps which are outlined briefly as follows:

1. We projected the population of Iran in 2025 in different scenarios.
2. We derived the age, sex, and disease-specific DALY rates from the first Iran’s National Burden of Disease (IRNBD) study in 2003 [12].
3. We applied the 2003 DALY rates [12] to the 2025 projected population under different scenarios to compute the expected DALY in 2025; Moreover, we subtracted the 2003 DALY from this estimation to provide a measure of the impact of demographic factors on the change in BOD between 2003 and 2025; we labeled this estimation as “DemBOD”. It is important to mention that 2003 DALY was computed based on the incidence based YLD.
4. We calculated the ratio of changes in number of death due to epidemiological and demographic components using the latest projection of GBD (Epi/Demo) [2]; then, we applied directly this ratio to the “DemBOD” to obtain a measure of the impact of epidemiological factors on the change in BOD between 2003 and 2025; this estimate was labeled as “EpiBOD”.
5. We summed up the estimates of “DemBOD” and “EpiBOD” to calculate the “total change” in BOD between 2003 and 2025.
6. We added the 2003 DALY to the “total change” to calculate the DALY in 2025. Detailed descriptions of these steps are given below:

### Population Projections

To obtain population projections, the Spectrum software (version 3.40) was applied. Nine scenarios were prepared by trends and changes in two factors: Total Fertility Rate (TFR) as a measure of fertility and Life Expectancy (LE) as a measure of mortality. For each of these two factors three probable scenarios were assumed (low, medium, and high TFR and LE).

The reported values for TFR and LE in 2010 according to the WHO latest report on World Health Statistics [13] and the United Nations Population Division (UNPD) projections [14] were used as main input data for 2010, in all three scenarios, to prepare consistent population projections. Their values for 2025 were assumed as follows: for the medium scenario no limitations were taken into account for 2025 and the software assumptions were accepted. For the other two scenarios, the TFR and LE were assumed to have approximately ±0.5 children per women and ±2 years change from their corresponding values in the medium scenario for 2025, respectively. Thus the final values in low, medium, and high scenarios in 2025 for TFR were 1.3, 1.8, and 2.3 children per women. These values for LE in men were 71.3, 73.3, and 75.3 years, and in women 74.7, 76.7, and 78.7, respectively.

For the purpose of population projections, we assumed the political and economical situation of Iran and its surrounding countries to be stable, with minimum impact of natural disasters.

### DALY Rates and Classification of Broad Cause Groups

The DALY rates per 100,000 population for each main cause group were separately extracted for men and women (classified in eight age groups) from IRNBD in 2003 [12]. In each age-sex category, these rates were classified into four broad cause groups based on the latest projection of GBD [2]. Hence, in this projection cause groups were as follows: 1) HIV/AIDS, 2) Group I: communicable diseases except HIV/AIDS, 3) Group II: non-communicable diseases (NCDs), and 4) Group III: Injuries.

For each demographic scenario, the age-, sex-, and broad cause- specific DALY rates were applied to the age- and sex- projected population and DALY in 2025 expected only on changing demography was obtained; then, the average value of each cause group specific DALY was obtained in the total population in 2025 across all demographic scenarios.

To project the DemBOD attributable to each cause group, the 2003 DALY of each cause group was subtracted from its corresponding average value in 2025 across all demographic scenarios.

This projection was based on the assumption that age-sex-specific DALY rates observed in 2003, will remain constant for the entire projection period (from 2003–2025).

### Projecting the Impact of Epidemiological Factors: Main Scenarios

We used the impact of income on the BOD to project the EpiBOD as well as 2025 DALY and considered two income scenarios, based on the World Bank’s country classification. As Iran’s income category has been recently upgraded, we considered the World Bank’s 2009 and 2012 classifications as probable scenarios. Moreover, we assumed the most likely apt scenario to estimate Iran’s BOD to be the one when following its past peer countries (lower-mid income countries) [15]–[16].

We separately calculated the Epi/Demo ratio for each cause group by different economic scenarios [2]. For each cause group by probable economic scenarios, we first applied Epi/Demo to the DemBOD to project the EpiBOD. We summed the EpiBOD and DemBOD to project the total change in BOD from 2003 to 2025 afterwards. Finally, the 2003 DALY were added to their corresponding total change to project DALY in 2025.

In this approach, we assumed the Epi/Demo ratio to be time-independent and directly applicable for possible changes in BOD. Moreover, it was assumed that Iran’s BOD will follow a similar pattern to that of the economic category.

We carried out all above steps on each cause group, but at the end of each step aggregated them into one group (called total), for presentation of the results. Moreover, all estimations were carried out per 100,000 population as well.

### Uncertainty Analysis

To evaluate the uncertainties in BOD, Monte Carlo method was employed using Stata (Version 10 for Windows). For full description of the modeling approach, please see Document S1.

## Results

### Trend of DemBOD between 2003 and 2025

Our population projections in 2025 foresee a substantial population increase over the next two decades. The projected population under the HH scenario (high TFR and high LE) was 90.7 million, compared with the LL scenario (low TFR and low LE) of 83.1 million, showing a difference of 8.3 percent; nearly 11% of population was more than 60 years in both scenarios (Table S1). We observed that the DemBOD will be comparable across all demographic scenarios in total (BOD in all four broad cause groups) and in all cause groups, except Group I. In total population, the lowest and highest values were in LL and HH scenarios, respectively. The DemBOD attributed to Group I followed a different pattern in different scenarios – It will increase in high TFR scenarios and decrease in medium and low TFR scenarios (Table S2).

**Figure 1:**
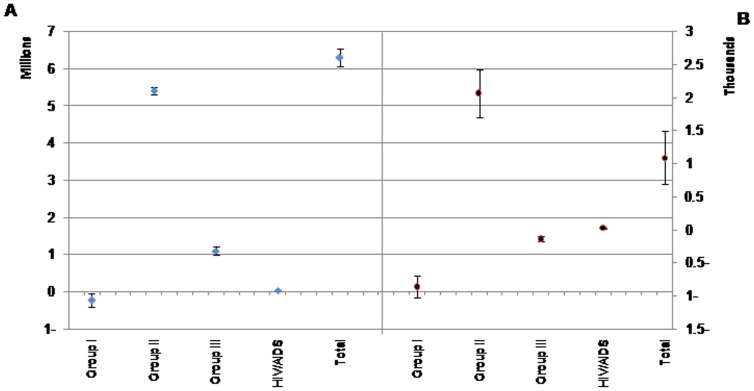
Projected the changes in disease burden due to demographic variations in broad cause groups and total across all demographic scenarios from 2003–2025. Results are classified in total population (A) and per 100,000 population (B). Vertical bars show the 95% uncertainty interval.

Our results showed that the average DemBOD is projected to increase in total and in all cause groups, except Group I. The total DemBOD will increase 6.3 million DALY (95% uncertainty interval [UI]: 6.1, 6.5). Regarding DemBOD attributed to Group II, III, and HIV/AIDS, our projections showed an increase of 5.41, 1.10, and 0.04 million DALY, respectively. However, we found a decrease of 0.24 million DALY in DemBOD attributed to Group I (Figure 1).

Per 100,000 population, the DemBOD attributed to Group I and III are projected to decrease (863 and 140 person-year, respectively), while the DemBOD attributed to Group II and HIV/AIDS are projected to increase (2066 and 21 person-year, respectively). The DemBOD in total will increase 1085 person-year (95% UI: 692, 1501).

### Trend of EpiBOD between 2003 and 2025

**Figure 2:**
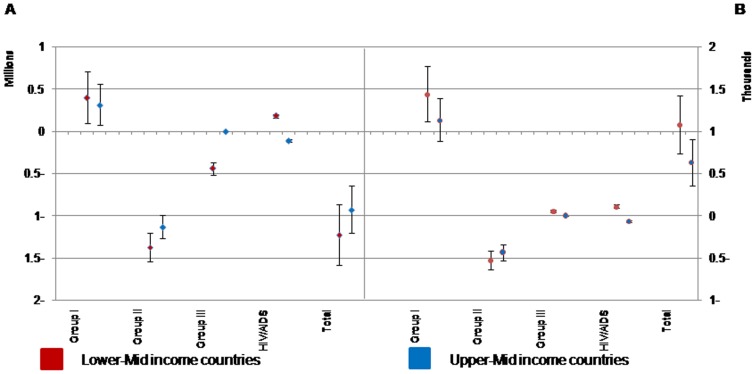
Projected the changes in disease burden due to epidemiological components in broad cause groups and total. Results are classified by Lower-Mid and Upper-Mid income scenarios from 2003 to 2025 in total population (A) and per 100,000 population (B). Vertical bars show the 95% uncertainty interval.

Following the economical pattern of lower-mid income countries versus the upper-mid income countries, the impact of EpiBOD will be more prominent in all cause groups and total. The projected total BOD due the epidemiological variations will decrease over time (between 0.93 and 1.23 million DALYs) (Figure 2).

We observed that in lower-mid- and upper-mid- income scenarios EpiBOD attributed to Groups I is projected to increase (0.39 and 0.31 million DALYs, respectively); while the EpiBOD attributed to Group II is projected to decrease (1.37 and 1.13 million DALYs, respectively).

The EpiBOD due to HIV/AIDS followed a different pattern; it will increase 183,000 DALYs in lower-mid income scenario, and decrease 110,000 DALYs in upper-mid income scenario. The BOD attributed to Group III is projected to decrease (0.44 million DALYs) in lower-mid income scenario and remain constant in upper-mid income scenario.

Following the lower-mid income scenario, in 100,000 population, the projected EpiBOD will decrease in Group II (524 person-year), and increase in Groups I, Group III, HIV/AIDS, and total (1439, 56, 110, and 1080 person-year, respectively).

Following the upper-mid income scenario, in 100,000 population, the EpiBOD will decrease in Group II and HIV/AIDS (433 and 65 person-year, respectively), and increase in total and Group I (629 and 1129 person-year, respectively), and remain constant in Group III.

### BOD in 2025 and its Changes from 2003

**Figure 3:**
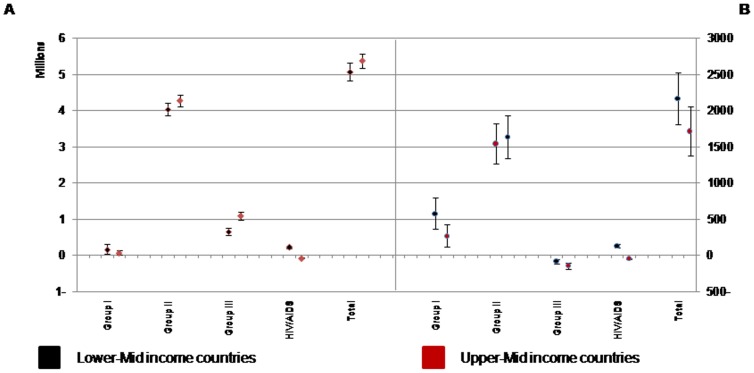
Projected the total changes in disease burden in broad cause groups and total. Results are classified by Lower-Mid and Upper-Mid income scenarios from 2003 to 2025 in total population (A) and per 100,000 population (B). Vertical bars show the 95% uncertainty interval.

Our projection scenarios in forecasting the total changes in BOD showed that the burden will increase in both lower-mid- and upper-mid- income scenarios in total and all cause groups, except HIV/AIDS. On the contrary, the projection of HIV/AIDS burden showed different results; in the lower-mid income scenario HIV/AIDS burden will have an increase of 219,000 while in upper-mid income scenario will have a decrease of about 73,000 DALYs (Figure 3).

Per 100,000 population, the BOD attributed to Group III is projected to decrease; while the BOD attributed to Group I, Group II, and total are projected to increase in both income scenarios. However, HIV/AIDS burden will increase to 132 person-years in lower-mid income scenario and decrease to 44 in upper-mid income scenario (Figure 3).

Our results showed that the total BOD will increase 35.3% from 2003 to 2025 in the most likely scenario. In this scenario, the DemBOD will increase 43.9% and EpiBOD decrease 8.6%. The total BOD will have an increase of 10.0% per 100,000 population; the increasing impact of DemBOD and EpiBOD will be 5%. Table 1 demonstrates the ratio of changes in BOD in each four cause groups from 2003–2025 to their corresponding values in 2003 along with the contribution of DemBOD and EpiBOD.

**Table 1:** Projected ratio of the impact of demographic, epidemiological, and total change on disease burden by 2025 to DALY in 2003 classified by broad cause groups (The most probable scenario).

	In total population			Per 100000 population		
	Demographic components	Epidemiological components	Totalchanges	Demographic components	Epidemiological components	Total changes
Group I	−14.5	24.2	9.7	−34.9	58.1	23.2
Group II	62.4	−15.8	46.5	14.6	−3.7	10.9
Group III	27.6	−11.1	16.5	−2.4	0.9	−1.4
HIV/AIDS	63.9	319.5	383.4	25.6	128	153

**Figure 4:**
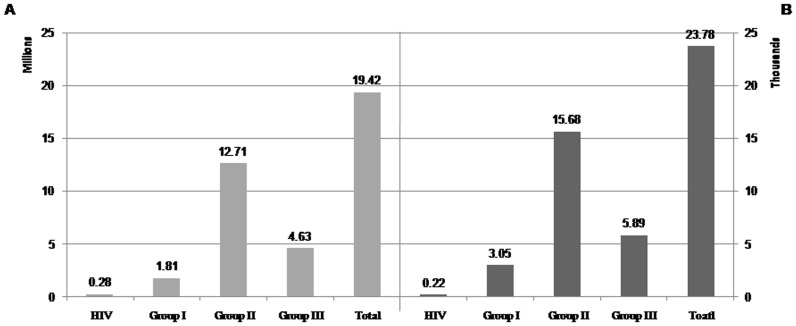
Projected the DALY in broad cause groups and total by 2025 (The most probable scenario). Panel A shows the 2025 BOD in total population and panel B per 100,000 population.

**Figure 5:**
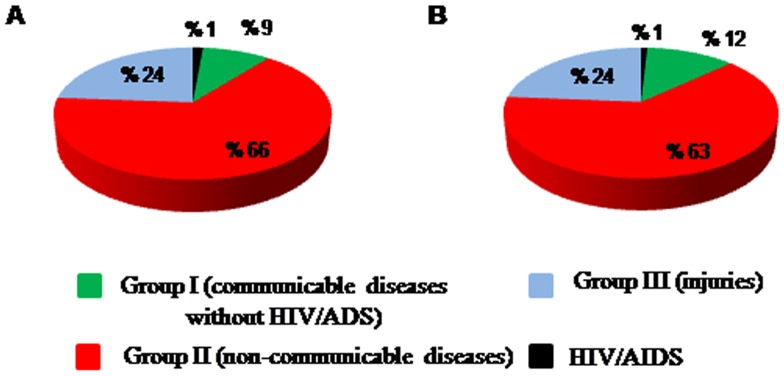
Projected proportional contribution of total disease burden by broad cause groups in 2025 (The most probable scenario). Panel A shows the values in total population and panel B per 100,000 population.

Total DALY due to demographic and epidemiological factors will increase from 14.35 million in 2003 to 19.42 million in 2025 (95% UI: 16.8.1, 21.9), an overall increase of 35.33%. The probability of losing one DALY will rise from 0.21 in 2003 to 0.24 in 2025 (an overall increase of 3%). The projection of DALY attributed to each broad cause group showed that Group II will be the leading cause group for the BOD, and account for about 66% of total BOD in 2025 (Figure 4 and 5).

## Discussion

Having used the best available data, we projected the BOD in Iran by 2025. Our projections showed that the DALY for all diseases and injuries will be 19.42 millions in 2025, predicting an increase of 35.332% compared to the estimates of 2003.We estimated that NCDs, injuries, and communicable diseases (except HIV/AIDS) will be the leading cause of losing healthy life. By comparison, the latest projection of GBD showed an increase of 3% in worldwide BOD in compare with the year 2002 [2].

The burden attributable to HIV/AIDS will considerably increase to 280,000 in 2025, which is five times greater than the estimate of 2003; its contribution will increase from 0.4% (in 2003) to 1% (in 2025). Compatible with our findings, studies have shown that HIV/AIDS will be one of the leading causes of DALY in middle income countries by 2030 [2]. In Iran, the number of new HIV cases has been on an upward trend since 1990 and it was projected to increase until 2012; moreover, it was projected that the number of HIV infected people will increase by the end of 2014 [17].

Our projections forecasted that the attributed BOD to Group I will have a slight increase by 2025. It was about 1.6 million DALYs in 2003 and will increase to 1.8 million DALY in 2025. Its contribution to the total DALY will decrease 2.5% by 2025 (its contribution to BOD was 11.5% in 2003).Other studies have also projected that the burden of this group will decrease in the upcoming years due to a recent decline in age-specific death rates [2], [4].

The burden due to Group II in 2025 is projected to be 1.5 times greater than that in 2003 (8.7 million) mainly due to aging of population. Moreover, its contribution to total BOD will be 5% greater than that of 2003. It has been projected that the worldwide BOD due to NCDs would increase from 50% (in 2002) to 57% (in 2030) [2]. Generally, most of NCDs such as cardio-vascular diseases, cancers, and mental and behavioral disorders are real health concerns nowadays and they have an increasing trend in most part of the world [18]. Therefore, it seems health systems have to develop long term and comprehensive plan to deal with this situation.

The Group III disease burden is projected to increase from 3.97 (in 2003) to 4.63 million (in 2025). However, its contribution to total BOD will decrease from 27.6% to 24.0% between 2003 and 2025 due to a major increase in the burden due to NCDs. Other studies have also projected that this group’s burden will increase, mostly because of the increasing number of deaths due to road traffic accidents as a result of economic growth in low and mid-income countries [2], [4].

Our projection suggests that demographic components will increase the DemBOD in all groups, except Group I. Their maximum and minimum increasing impacts were observed in the HH and LL scenarios, respectively. It seems that aging has minimum impact in increasing DemBOD in different TFR scenarios and maximum DemBOD was observed in scenarios with high life expectancy. However, the changes in TFR have more or less comparable impact on DemBOD in different LE scenarios. The finding showed a positive association between TFR and DemBOD.

Regarding the impact of epidemiological components, we projected that the changes in these factors will decrease the BOD. Iran’s economic growth in recent decades is undeniable [11]. This is associated with increasing the level of development through education, changing people’s lifestyles, advances in medical technology and improving preventive healthcare systems. We believe that chronic diseases onset and their complications as well as the disability burden accordingly could be decreased or delayed. If these changes continue, it is probable that the amount of decrease might be more than that of projected here.

Having compared the impact of EpiBOD and DemBOD, we found that they will act in opposite directions. HIV/AIDS is however an exception, in which both epidemiological and demographic components will increase its burden. Further findings suggest that the greatest increasing impact of demographic and epidemiological components may be observed in Group II and HIV/AIDS, respectively.

According to WHO report, the LE of Iranian men has increased from 61 years in 1989 to 70 years in 2009; the corresponding numbers for women were 65 years and 75 years [13], [19]. UNPD projects LE of 73.3 and 77.3 years in men and women by 2025, respectively [14]. Therefore, the impact of aging on BOD will be comparable in both sexes. Moreover, recent figures have shown a downward trend in TFR from five children per women in 1989 to 1.7 in 2010 [13], [19]. The UNPD data bank also shows a decrease in the population growth rate in a way that it is estimated to reach 0.51 by 2025 [14]. It seems that older Iranian populations, due to longer LE and low TFR, will generate more disability but less premature death; which is comparable with the pattern in other countries [2], [4]–[6], [16], [20].

These projections have been prepared using a simple approach, with the least information available. In order to deal with the shortage of information we assumed that the changes of epidemiological components in Iran will follow its pattern in other countries with similar socio-economical level. For this purpose two scenarios were created, compatible with lower and upper middle income countries.

Due to some limitations in the projection method, these estimates should be interpreted with caution. The lack of historical trend information (in risk factors, death rates, and DALY) made us use the trends of other countries with similar socio-economical level. However, we took these uncertainties into account in our Monte-Carlo analysis.

In addition, disaggregation of DALY by YLL and YLD was not assessed due to lack of valid long-term information about the mortality and morbidity, incidence and prevalence of main diseases in Iran. Besides, baseline DALY rates in this study were computed using “incidence YLD” in 2003, hence, our results may need adjustments in order to be used for health policy and planning. Since this study was the first of its kinds in Iran and due to scarcity of data, we could not assess the trend of BOD in different diseases such cancers, cardio-vascular diseases, or respiratory diseases, etc. However, it is very important issue which has to be addressed in future studies.

It seems that the BOD will increase around 35% in Iran in the next two decades, mainly due to aging. Our model shows that NCDs will be the leading cause of DALY in 2025 in Iran; however, the increasing trend of BOD due to HIV/AIDS will be important. As might be expected, we found that BOD due to Group I (other infectious diseases, malnutrition, infant and maternal mortality rates) will have a very slight increase.

## Supporting Information

Table S1 **Projected population size and the proportion of adults over 60 years in Iran in 2025 by different demographic scenarios.**
(DOC)

Table S2 **Projected the DemBOD in broad cause groups and total from 2002–2025.** (Results are classified by different scenarios of the life expectancy and Total Fertility Rate in total population and per 100,000 population).
(DOC)

Document S1 **Uncertainty analysis.**
(DOC)
