# Supplementary material for: The Projection of Burden of Disease in Islamic Republic of Iran to 2025
Source: PLoS One. 2013 Oct 17;8(10):e76881. doi: 10.1371/journal.pone.0076881 (PMC3798284; doi:10.1371/journal.pone.0076881)
Supplement: Table S1 — Projected population size and the proportion of adults over 60 years in Iran in 2025 by different demographic scenarios. (DOC) [file pone.0076881.s001.doc]

**Table S1: Projected population size and the proportion of adults over 60 years in Iran in 2025 by different demographic scenarios**

| **Demographic scenarios** | **Projected population size** | **Fraction of 60≥ years old population** |
| --- | --- | --- |
| **LL** (low TFR & low LE) | 83,154,785 | 11.94 |
| **LM** (low TFR & medium LE) | 83,230,542 | 11.97 |
| **LH** (low TFR & high LE) | 83,288,290 | 11.99 |
| **ML** (medium TFR & low LE) | 86,870,984 | 11.42 |
| **MM** (medium TFR & medium LE) | 86,949,693 | 11.46 |
| **MH** (medium TFR & high LE) | 87,009,317 | 11.49 |
| **HL** (high TFR & low LE) | 90,587,184 | 10.96 |
| **HM** (high TFR & medium LE) | 90,668,844 | 10.99 |
| **HH** (high TFR & high LE) | 90,725,387 | 11.01 |

TFR: Total Fertility Rates; LE: Life Expectancy
