# Supplementary material for: The Projection of Burden of Disease in Islamic Republic of Iran to 2025
Source: PLoS One. 2013 Oct 17;8(10):e76881. doi: 10.1371/journal.pone.0076881 (PMC3798284; doi:10.1371/journal.pone.0076881)
Supplement: Table S2 — Projected the DemBOD in broad cause groups and total from 2002–2025. (Results are classified by different scenarios of the life expectancy and Total Fertility Rate in total population and per 100,000 population). (DOC) [file pone.0076881.s002.doc]

**Table S2: Projected the DemBOD in broad cause groups and total from 2002-2025. (Results are classified by different scenarios of the life expectancy and Total Fertility Rate in total population and per 100,000 population)**

|  | | HIV | Group I | Group II | Group III | total |
| --- | --- | --- | --- | --- | --- | --- |
| **In total population** | | | | | | |
| **LL** | 36,381.07 | | -515,000.11 | 5,259,210.84 | 930,725.12 | 5,711,316.92 |
| **LM** | 36,451.18 | | -513,495.30 | 5,283,127.43 | 937,179.78 | 5,743,263.10 |
| **LH** | 36,476.37 | | -512,398.73 | 5,295,130.84 | 940,762.97 | 5,759,971.46 |
| **ML** | 36,472.32 | | -240,571.09 | 5,387,303.81 | 1,091,637.65 | 6,274,842.68 |
| **MM** | 36,542.48 | | -238,627.90 | 5,411,342.83 | 1,098,232.64 | 6,307,490.05 |
| **MH** | 36,567.70 | | -237,167.76 | 5,423,431.58 | 1,101,914.17 | 6,324,745.69 |
| **HL** | 36,563.57 | | 33,857.90 | 5,515,396.78 | 1,252,550.14 | 6,838,368.38 |
| **HM** | 36,631.31 | | 36,230.55 | 5,539,093.08 | 1,259,175.34 | 6,871,130.27 |
| **HH** | 36,653.28 | | 37,959.43 | 5,550,650.22 | 1,262,633.22 | 6,887,896.15 |
| **Per 100,000 population** | | | | | | |
| **LL** | | 26.602 | -1,114.741 | 2,611.964 | -76.322 | 2515.003 |
| **LM** | | 26.564 | -1,114.422 | 2,622.371 | -75.016 | 2526.996 |
| **LH** | | 26.536 | -1,113.800 | 2,628.234 | -73.721 | 2534.749 |
| **ML** | | 21.895 | -857.091 | 2,042.687 | -143.298 | 2131.693 |
| **MM** | | 21.859 | -856.606 | 2,052.836 | -142.014 | 2143.574 |
| **MH** | | 21.833 | -855.754 | 2,058.476 | -140.754 | 2151.301 |
| **HL** | | 17.573 | -620.580 | 1,520.118 | -204.779 | 1779.833 |
| **HM** | | 17.540 | -619.909 | 1,529.828 | -203.519 | 1791.439 |
| **HH** | | 17.515 | -618.886 | 1,535.115 | -202.451 | 1798.793 |
