# Supplementary material for: The Projection of Burden of Disease in Islamic Republic of Iran to 2025
Source: PLoS One. 2013 Oct 17;8(10):e76881. doi: 10.1371/journal.pone.0076881 (PMC3798284; doi:10.1371/journal.pone.0076881)
Supplement: Document S1 — Uncertainty analysis. (DOC) [file pone.0076881.s003.doc]

**Document S1:** Uncertainty analysis

In order to evaluate the uncertainties in our estimates for the total population and similarly per 100,000 population, we proceeded as follows for each broad cause group:

1. **DemBOD:** A random sample of size 10,000 generated from DALY rates in 2025 (expected only on demographic variations); by assuming the average value and standard deviation across all demographic scenarios as the mean and standard error of the distribution (sample 1). The 2003 DALY rate from IRNBD was subtracted from sample 1 to calculate DemBOD (sample 2).
2. **EpiBOD:** First, in each income group, a random sample size of 10,000 generated from the distribution of Epi/Demo ratio; by assuming this value as the mean distribution and applying ±20% change to calculate its standard error (sample 3). In each income group, by multiplying sample 2 and 3, a random sample size of 10,000 was provided for EpiBOD (sample 4).
3. **Total changes in BOD:** Were calculated by adding up samples 2 and 4 (called sample 5).
4. **DALY in 2025:** The DALY rate from IRNBD was added to its corresponding total change up to 2025 (i.e. sample 5) and the DALY in 2025 was obtained (called sample 6).

At the end of each step, the 2.5^th^ and 97.5^th^ percentiles of the distribution were considered as the lower and upper bound of the 95% uncertainty interval. Moreover, the BOD in total was calculated by adding the values for all four broad causes and its 95% uncertainty interval was obtained in the same way.

Full mathematical descriptions of the above approach as well as our approach in calculating DALY can be obtained from the author upon request.
